# Construction and evaluation of a 180-day readmission prediction model for chronic heart failure patients based on sCD40L

**DOI:** 10.1097/MD.0000000000042134

**Published:** 2025-04-11

**Authors:** Peng Zhang, Xiaoya Zhai, Yefei Gao, Liping Meng, Hui Lin, Ping Wang, Chengjian Jiang

**Affiliations:** a Department of Cardiology, Shaoxing People’s Hospital, Shaoxing, Zhejiang, China.

**Keywords:** chronic heart failure, COX regression, prediction model, readmission, sCD40L

## Abstract

The high readmission rate of patients with chronic heart failure (HF) can cause waste of medical resources and economic losses. Establishing an effective HF readmission model can effectively alleviate medical pressure and improve the quality of treatment. In this study, we conducted a comprehensive analysis of clinical and laboratory data from 248 patients with chronic HF who received treatment at our medical center between January 2021 to January 2022. We also measured soluble CD40 ligand (sCD40L) levels to determine their association with readmission due to HF during follow-up. To analyze the data, we employed various statistical methods including one-way ANOVA, correlation analysis, univariate COX regression, and Least Absolute Shrinkage and Selection Operator COX regression. Using these techniques, we organized the data and constructed a predictive model that was both trained and validated. We developed a nomogram to assess the likelihood of readmission within 180 days for patients with chronic HF. Our findings revealed that monocytes, creatinine, sCD40L, and hypertension history were all independent risk factors for 180-day HF readmissions. Additionally, our model’s AUC was 0.731 in the training dataset and 0.704 in the validation dataset. This study provides new insights for predicting readmission within 180 days for patients with chronic HF. And sCD40L is an important predictive indicator for readmission of HF patients within 180 days, and clinical doctors can develop appropriate treatment plans based on sCD40L.

## 1. Introduction

Heart failure (HF) is a major cause of morbidity and mortality globally.^[1]^ Large-scale data indicate an increasing prevalence of HF, high hospitalization costs, and increased frequency of hospitalization, which places a huge burden on both individual and social healthcare and may become a more serious challenge in the future.^[2]^ Many people worldwide have high blood pressure, diabetes, obesity, kidney disease, and other risk factors for heart disease.^[3]^ Some researchers have estimated that the incidence of HF will continue to increase over the coming decades.^[2]^

Currently, HF is difficult to treat, and most patients require readmissions. This requires considerable time, money, and energy. Therefore, a prediction model for HF readmission that fits the characteristics of clinical practice is needed. Studies predicting the risk of readmission in HF patients have been based on clinical parameters such as brain natriuretic peptide or N-terminal brain natriuretic peptide levels, renal insufficiency, comorbidities, and previous hospitalization history.^[4]^ However, in terms of “risk prediction,” classic cardiovascular risk markers such as soluble CD40 ligand (sCD40L), have rarely been explored.

As a key immune factor, sCD40L is primarily expressed in immune cells^[5]^ and activated platelets,^[6]^ and its role in vascular inflammation cannot be ignored.^[7]^ Many studies have confirmed that the serum expression level of sCD40L is positively correlated with cardiovascular events, and higher sCD40L levels are associated with coronary microvascular dysfunction.^[8]^ Some studies have suggested that sCD40L can be used as a diagnostic marker for systemic atherosclerosis.^[9]^ Clinical studies have shown that acute or chronic HF patients have elevated sCD40L levels.^[10]^ In addition, few studies have evaluated the risk of readmission in chronic HF patients based on sCD40L levels. Therefore, an analysis of readmission risk in HF patients based on sCD40L expression levels may provide a better risk assessment in a clinical setting.

## 2. Method

### 2.1. Study population

**Figure 1.**
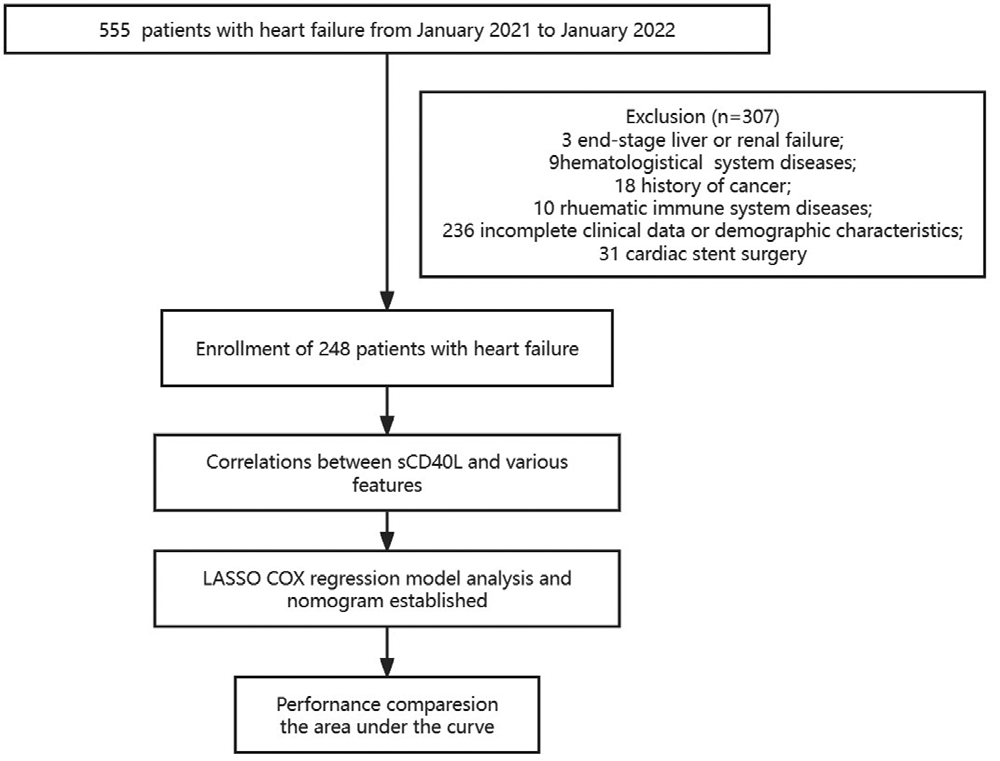
Workflow diagram of the study.

This study included chronic HF patients who visited the Cardiology Department of Shaoxing People’s Hospital between January 2021 and January 2022. All patients are inpatients. The inclusion criteria were as follows: (1) age > 18 years; (2) patients diagnosed to have chronic HF based on the guidelines of the European Heart Association, and patients with extent of HF between class II and IV according to the New York Heart Association; (3) those with a history of congestive HF for at least a month. Patients with the following conditions were excluded: (1) end-stage liver or renal failure; (2) hematological system diseases; (3) history of cancer or tumor resection; (4) rheumatic immune system diseases; (5) those with incomplete clinical data or demographic characteristics; (6) patients readmitted to other hospitals; and (7) those who have undergone cardiac stent surgery (Fig. 1). Demographic and clinical data, as well as patient blood samples, were collected on the second day of hospitalization. The date of the patient’s discharge is recorded as the date of enrollment. Information on readmission of the patient is recorded through a telephone follow-up 180 days later. A total of 248 patients who met the inclusion criteria were included in the study. The study was conducted in accordance with the Declaration of Helsinki and approved by the Hospital Ethics Review Committee.

### 2.2. Clinical and laboratory data

All blood samples were taken within 1 to 2 days after admission. Blood samples were obtained via venous puncture and collected in pre-cooled tubes containing ethylene diamine tetraacetic acid. The supernatant was centrifuged and stored at -80 °C. According to the manufacturer’s protocol, an enzyme-linked immunosorbent assay kit (ab196268, Abcam) was used to measure sCD40L levels.

### 2.3. Clinical outcomes

The date of the patient’s discharge is recorded as the date of enrollment. Information on readmission of the patient is recorded through a telephone follow-up 180 days later. The main clinical outcome was that patients were admitted due to worsening chronic HF. The endpoint was determined by contacting each patient individually.

### 2.4. Statistical analysis

Continuous variables are represented as means and standard deviations, while categorical variables are represented as frequencies and percentages. A normality test was performed on the data distribution to select the appropriate test. Continuous symmetric variables were tested using an independent *t* test, and categorical variables were tested using the Pearson chi-square test (Pearson χ^2^ test). The Spearman correlation test was used to determine the correlations between various variables. Evaluate the diagnostic ability of each parameter or model by analyzing the area under the curve (AUC) of the receiver operator characteristic curve analysis.

The initial dataset has been randomly divided into training and validation datasets in a 7:3 ratio to create and validate a prediction model. The Least Absolute Shrinkage and Selection Operator (LASSO) method has been used to select meaningful indicators from the training dataset, followed by COX regression to establish the prediction model and construct a nomogram. A calibration curve was constructed in the training group to predict the similarity between the predicted probability and the actual observation probability. In addition, decision curve analysis evaluates the clinical practicality of the column chart by quantifying the net benefits under different threshold probabilities.

The R version 4.1.0 (The R Foundation for Statistical Computing, Vienna, Austria) was used for all data analyses and models. All statistical tests were two-tailed. *P* < .05 is considered statistically significant.

## 3. Result

### 3.1. Baseline characteristics

The baseline characteristics of the study participants are shown in Table 1. We divided the readmission time of chronic HF patients into 4 subgroups according to quartiles and analyzed the relationship between readmission time and various indicators. Our results showed that patients with shorter readmission times had higher levels of serum creatinine (*P* = .036) and sCD40L (*P* = .004). Interestingly, the trend of patients’ diabetes history was opposite, with a lower incidence of diabetes being observed in patients with shorter readmission times. However, there was no statistically significant difference in other features.

**Table 1:** Patients characteristics.

Variables	First quartile (n = 62)	Second quartile (n = 62)	Third quartile(n = 61)	Fourth quartile(n = 63)	*P*-value
Sex					.442
Female	25 (40.3%)	20 (32.3%)	22 (36.1%)	17 (27.0%)	
Male	37 (59.7%)	42 (67.7%)	39 (63.9%)	46 (73.0%)	
Age, years	58.0 (9.70)	57.1 (12.1)	60.4 (13.6)	55.7 (14.0)	.19
WBC, ×10^12^/L	5.22 (1.69)	5.40 (2.24)	5.27 (1.62)	5.96 (2.33)	.141
Neutrophils, ×10^12^/L	5.51 (3.24)	7.05 (9.90)	5.20 (2.73)	6.00 (8.10)	.448
Lymphocyte, ×10^12^/L	4.35 (8.72)	4.76 (9.50)	4.59 (13.4)	4.77 (10.5)	.996
Monocyte, ×10^12^/L	2.66 (3.43)	2.80 (3.09)	2.44 (2.77)	1.89 (2.65)	.346
RBC, ×10^12^/L	3.17 (1.67)	3.31 (2.74)	3.14 (1.72)	3.81 (2.69)	.328
hsCRP, mg/L	2.94 (6.65)	4.35 (9.21)	3.71 (7.19)	3.83 (8.43)	.804
AST, U/L	26.1 (11.0)	338 (2395)	27.5 (12.2)	29.1 (19.9)	.374
LDH, U/L	230 (59.6)	495 (2009)	231 (79.1)	226 (107)	.358
Albumin, g/L	37.5 (3.95)	37.1 (5.38)	37.1 (4.87)	37.6 (5.30)	.917
Globulin, g/L	28.9 (4.27)	29.8 (4.50)	28.8 (4.10)	29.5 (5.31)	.597
Creatinine, µmol/L	86.8 (29.4)	109 (98.6)	106 (93.5)	77.0 (23.3)	**.036**
Urea, µmol/L	420 (115)	417 (137)	435 (142)	384 (130)	.18
TG, mmol/L	1.33 (0.71)	1.20 (0.68)	1.24 (0.66)	1.36 (0.73)	.534
HDL-C, mmol/L	1.08 (0.28)	1.08 (0.33)	1.05 (0.29)	1.04 (0.30)	.881
LDL-C, mmol/L	2.54 (0.70)	2.47 (0.85)	2.39 (0.73)	2.65 (0.81)	.268
Apo A1, g/L	1.13 (0.24)	1.09 (0.28)	1.09 (0.27)	1.09 (0.28)	.759
Apo B, g/L	0.84 (0.24)	0.85 (0.29)	0.81 (0.22)	0.91 (0.28)	.171
FBG, mmol/L	6.64 (3.54)	6.90 (3.64)	6.07 (2.36)	7.26 (3.41)	.228
ALT, U/L	26.5 (17.5)	120 (705)	27.9 (32.6)	33.0 (29.8)	.384
HBA1c, %	6.77 (1.76)	6.88 (1.63)	6.53 (1.36)	7.07 (2.06)	.366
sCD40L, ng/mL	9.65 (2.02)	9.64 (2.00)	10.1 (1.93)	8.76 (2.25)	**.004**
LVEF, %	49.1 (13.1)	49.7 (13.1)	47.3 (13.4)	53.5 (15.0)	.081
LVFS, %	25.6 (8.16)	26.5 (7.87)	25.0 (8.95)	28.5 (9.25)	.117
AF					.067
No	33 (53.2%)	31 (50.0%)	28 (45.9%)	43 (68.3%)	
Yes	29 (46.8%)	31 (50.0%)	33 (54.1%)	20 (31.7%)	
Hypertension					.087
No	28 (45.2%)	30 (48.4%)	26 (42.6%)	40 (63.5%)	
Yes	34 (54.8%)	32 (51.6%)	35 (57.4%)	23 (36.5%)	
Hyperlipidemia					.656
No	60 (96.8%)	58 (93.5%)	60 (98.4%)	60 (95.2%)	
Yes	2 (3.23%)	4 (6.45%)	1 (1.64%)	3 (4.76%)	
Diabetes					**.032**
No	43 (69.4%)	36 (58.1%)	45 (73.8%)	32 (50.8%)	
Yes	19 (30.6%)	26 (41.9%)	16 (26.2%)	31 (49.2%)	
Smoking					.473
No	48 (77.4%)	40 (64.5%)	43 (70.5%)	44 (69.8%)	
Yes	14 (22.6%)	22 (35.5%)	18 (29.5%)	19 (30.2%)	
Drinking					.171
No	54 (87.1%)	47 (75.8%)	51 (83.6%)	46 (73.0%)	
Yes	8 (12.9%)	15 (24.2%)	10 (16.4%)	17 (27.0%)	
SBP, mm Hg	136 (19.9)	131 (20.4)	132 (23.6)	127 (20.8)	.153
DBP, mm Hg	83.7 (15.4)	80.0 (13.4)	80.6 (13.4)	78.7 (13.6)	.238
BMI, kg/m^2^	24.1 (3.80)	23.4 (2.82)	23.8 (3.70)	24.3 (3.82)	.491
Readmission day	36.2 (14.3)	80.4 (14.2)	131 (16.3)	275 (130)	**<.001**

Variables with normal distribution were presented as mean ± standard deviation, other variables with classification were described as counts (percentages).

Discrepant *P*-values are represented in bold.

AF = atrial fibrillation, ALT = Alaninetransaminase, Apo A1 = Apolipoprotein A1, Apo B = Apolipoprotein B, AST = aspartate amino transferase, BMI = body mass index, DBP = diastolic blood pressure, FBG = fasting blood glucose, HBA1c = hemoglobin A1c, HDL-C = high-density lipoprotein cholesterol, hsCRP = highly sensitive C-reactive protein, LDH = lactate dehydrogenase, LDL-C = low-density lipoprotein cholesterol, LVEF = left ventricular ejection fraction, LVFS = left ventricular fractional shortening, RBC = red blood cell, SBP = systolic blood pressure, sCD40L = soluble CD40 ligand, TG = triglyceride, WBC = white blood cell.

### 3.2. Correlation between sCD40L and various features

**Figure 2.**
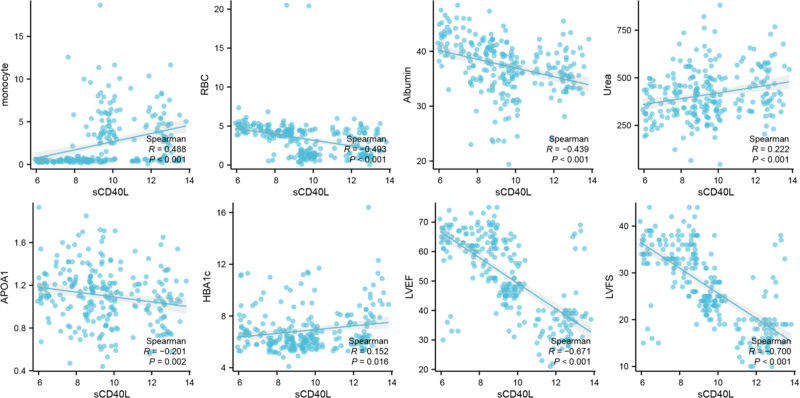
Correlation between sCD40L and various features. sCD40L = soluble CD40 ligand.

As a novel biomarker, sCD40L may be associated with the patient’s readmission time and the severity of HF. Therefore, we aimed to investigate the correlation between sCD40L and various clinical features. We divided chronic HF patients into 4 subgroups based on quartile values of sCD40L concentration (Table 2). Our results revealed significant differences between sCD40L and several serological indicators including white blood cells (*P* < .001), monocytes (*P* < .001), and red blood cell (RBC) (*P* < .001). Additionally, sCD40L showed association with several blood biochemical indicators such as albumin (*P* < .001), blood uric acid (*P* = .002), low-density lipoprotein cholesterol (*P* = .038), Apolipoprotein A1 (*P* = .018), Apolipoprotein B (*P* = .004), and left ventricular ejection fraction (LVEF) (*P* < .001). In terms of demographic data, sCD40L was found to be related to the history of atrial fibrillation (*P* < .001), hypertension (*P* = .001), diabetes (*P* < .001), and alcohol consumption (*P* = .037). Interestingly, we observed a negative correlation between sCD40L and fasting blood glucose (*P* < .001) as well as hemoglobin A1c (HBA1C) (*P* = .001). Based on the results of relevance, we conducted a correlation analysis scatter plot for continuous variables with a *P*-value <.05 and sCD40L. As shown in Figure 2, there is a significant correlation between monocyte, RBC, serum albumin, urea, Apolipoprotein A1, HBA1c, LVEF, left ventricular fractional shortening (LVFS), and sCD40L. Among them, the correlation between LVFS and sCD40L is the most obvious. This suggests that sCD40L might have some kind of association with the severity of HF in patients.

**Table 2:** Baseline characteristics of patients according to quartile levels of sCD40L.

Variables	First quartile (n = 62)	Second quartile (n = 63)	Third quartile(n = 61)	Fourth quartile(n = 62)	*P*-value
Sex					
Female	21 (33.9%)	30 (47.6%)	18 (29.5%)	15 (24.2%)	**.038**
Male	41 (66.1%)	33 (52.4%)	43 (70.5%)	47 (75.8%)	
Age, years	52.4 (11.8)	60.2 (13.8)	61.2 (11.6)	57.3 (11.0)	**<.001**
WBC, ×10^12^/L	6.31 (2.40)	5.60 (1.68)	4.77 (2.08)	5.16 (1.45)	**<.001**
Neutrophils, ×10^12^/L	5.43 (9.98)	5.88 (8.27)	6.60 (2.84)	5.86 (2.59)	.816
Lymphocyte, ×10^12^/L	3.51 (11.9)	2.86 (6.69)	7.29 (14.2)	4.89 (7.61)	.096
Monocyte, ×10^12^/L	0.75 (1.69)	1.88 (3.31)	4.00 (2.86)	3.19 (2.86)	**<.001**
RBC, ×10^12^/L	4.54 (1.14)	3.84 (2.52)	2.33 (2.71)	2.70 (1.66)	**<.001**
hsCRP, mg/L	1.81 (1.89)	5.97 (10.0)	3.95 (10.2)	3.07 (5.96)	.026
AST, U/L	27.7 (21.3)	31.0 (27.1)	338 (2415)	29.4 (13.4)	.386
LDH, U/L	200 (88.6)	242 (83.5)	501 (2025)	244 (80.4)	.33
Albumin, g/L	40.6 (4.48)	37.4 (4.91)	35.0 (4.57)	36.3 (3.74)	**<.001**
Globulin, g/L	29.6 (4.52)	29.4 (4.75)	29.3 (4.70)	28.6 (4.32)	.674
Creatinine, µmol/L	75.1 (32.0)	95.9 (69.9)	103 (88.2)	104 (79.2)	.087
Urea, µmol/L	376 (106)	417 (144)	399 (137)	462 (125)	**.002**
TG, mmol/L	1.63 (0.92)	1.11 (0.55)	1.21 (0.59)	1.18 (0.54)	**<.001**
HDL-C, mmol/L	1.07 (0.28)	1.11 (0.34)	1.05 (0.30)	1.03 (0.26)	.56
LDL-C, mmol/L	2.72 (0.79)	2.37 (0.67)	2.38 (0.80)	2.58 (0.81)	**.038**
Apo A1, g/L	1.17 (0.25)	1.14 (0.28)	1.06 (0.28)	1.04 (0.24)	**.018**
Apo B, g/L	0.94 (0.29)	0.78 (0.22)	0.82 (0.23)	0.87 (0.27)	**.004**
FBG, mmol/L	8.21 (4.15)	5.86 (2.76)	6.65 (3.28)	6.17 (2.25)	**<.001**
ALT, U/L	30.3 (27.9)	30.2 (32.6)	117 (711)	30.7 (29.6)	.429
HBA1c, %	7.54 (2.43)	6.33 (1.26)	6.72 (1.54)	6.65 (1.18)	**.001**
sCD40L, ng/mL	6.95 (0.67)	8.75 (0.37)	9.94 (0.49)	12.5 (0.59)	**<.001**
LVEF, %	64.4 (4.36)	57.8 (9.77)	44.0 (4.92)	33.2 (5.35)	**<.001**
LVFS, %	35.1 (3.34)	31.1 (6.40)	22.3 (2.71)	17.0 (6.06)	**<.001**
AF					**<.001**
No	48 (77.4%)	22 (34.9%)	30 (49.2%)	35 (56.5%)	
Yes	14 (22.6%)	41 (65.1%)	31 (50.8%)	27 (43.5%)	
Hypertension					**.001**
No	42 (67.7%)	25 (39.7%)	22 (36.1%)	35 (56.5%)	
Yes	20 (32.3%)	38 (60.3%)	39 (63.9%)	27 (43.5%)	
Hyperlipidemia					.159
No	60 (96.8%)	63 (100%)	57 (93.4%)	58 (93.5%)	
Yes	2 (3.23%)	0 (0.00%)	4 (6.56%)	4 (6.45%)	
Diabetes					**<.001**
No	20 (32.3%)	49 (77.8%)	45 (73.8%)	42 (67.7%)	
Yes	42 (67.7%)	14 (22.2%)	16 (26.2%)	20 (32.3%)	
Smoking					.143
No	44 (71.0%)	49 (77.8%)	45 (73.8%)	37 (59.7%)	
Yes	18 (29.0%)	14 (22.2%)	16 (26.2%)	25 (40.3%)	
Drinking					**.037**
No	49 (79.0%)	58 (92.1%)	45 (73.8%)	46 (74.2%)	
Yes	13 (21.0%)	5 (7.94%)	16 (26.2%)	16 (25.8%)	
SBP, mm Hg	130 (18.6)	134 (23.5)	135 (21.2)	126 (20.7)	.059
DBP, mm Hg	81.3 (11.8)	81.6 (14.9)	79.2 (12.6)	80.9 (16.5)	.801
BMI, kg/m^2^	24.0 (3.71)	24.2 (3.43)	23.0 (3.05)	24.4 (3.90)	.136
Readmission day	173 (151)	107 (65.8)	128 (120)	118 (83.1)	**.005**

Variables with normal distribution were presented as mean ± standard deviation, other variables with classification were described as counts (percentages).

Discrepant *P*-values are represented in bold.

AF = atrial fibrillation, ALT = Alaninetransaminase, Apo A1 = Apolipoprotein A1, Apo B = Apolipoprotein B, AST = aspartate amino transferase, BMI = body mass index, DBP = diastolic blood pressure, FBG = fasting blood glucose, HBA1c = hemoglobin A1c, HDL-C = high-density lipoprotein cholesterol, hsCRP = highly sensitive C-reactive protein, LDH = lactate dehydrogenase, LDL-C = low-density lipoprotein cholesterol, LVEF = left ventricular ejection fraction, LVFS = left ventricular fractional shortening, RBC = red blood cell, SBP = systolic blood pressure, sCD40L = soluble CD40 ligand, TG = triglyceride, WBC = white blood cell.

### 3.3. Feature construction and regression analysis

In univariate COX regression analysis, the following parameters were associated with readmission: age (odd radio (OR): 1.01; 95% confidence interval (CI): 1.00–1.02; *P* = .017), WBC (OR: 0.85; 95% CI: 0.78–0.91; *P* = .011), monocyte (OR: 1.07; 95% CI: 1.03–1.10; *P* < .001), RBC (OR: 0.88; 95% CI: 0.81–0.94; *P* < .001), albumin (OR: 0.95; 95% CI: 0.93–0.98; *P* < .001), creatinine (OR: 1.01; 95% CI: 1.00–1.01; *P* = .007), urea (OR: 1.01; 95% CI: 1.00–1.01; *P* = .002), triglyceride (OR: 0.75; 95% CI: 0.62–0.91; *P* = .003), Apolipoprotein B (OR: 0.49; 95% CI: 0.30–0.83; *P* = .007), HBA1c (OR: 1.18; 95% CI: 1.11–1.25; *P* < .001), LVEF (OR: 0.96; 95% CI: 0.95–0.97; *P* < .001), LVFS (OR: 0.94; 95% CI: 0.92–0.96; *P* < .001), history of atrial fibrillation (OR: 1.71; 95% CI: 1.31–2.23; *P* < .001), and history of hypertension (OR: 1.41; 95% CI: 1.10–1.83; *P* = .007) (Table 3).

**Table 3:** Univariate COX regression analysis.

Variable	Unadjusted HR (95%CI)	*P*-value
Sex		
Male	Reference	
Female	0.87 (0.66–1.13)	.288
Age	1.01 (1.00–1.02)	**.017**
WBC	0.85 (0.78–0.91)	**.011**
Neutrophils	1.01 (0.99–1.02)	.233
Lymphocyte	1.00 (0.99–1.02)	.268
Monocyte	1.07 (1.03–1.10)	**<.001**
RBC	0.88 (0.81–0.94)	**<.001**
hsCRP	1.01 (0.99–1.02)	.202
AST	1.00 (0.99–1.00)	.432
LDH	1.00 (0.99–1.00)	.317
Albumin	0.95 (0.93–0.98)	**<.001**
Globulin	1.00 (0.97–1.03)	.77
Creatinine	1.01 (1.00–1.01)	**.007**
Urea	1.01 (1.00–1.01)	**.002**
TG	0.75 (0.62–0.91)	**.003**
HDL-C	1.04 (0.67–1.60)	.875
LDL-C	0.85 (0.72–1.01)	.07
Apo A1	0.79 (0.49–1.27)	.326
Apo B	0.49 (0.30–0.83)	**.007**
FBG	0.97 (0.89–1.06)	.097
ALT	1.00 (0.99–1.00)	.491
HBA1c	1.18 (1.11–1.25)	**<.001**
LVEF	0.96 (0.95–0.97)	**<.001**
LVFS	0.94 (0.92–0.96)	**<.001**
AF		
No	Reference	**<.001**
Yes	1.71 (1.31–2.23)	
Hypertension		
No	Reference	**.007**
Yes	1.41 (1.10–1.83)	
Hyperlipidemia		
No	Reference	
Yes	0.96 (0.50–1.80)	.887
Diabetes		
No	Reference	
Yes	0.85 (0.37–1.33)	.394
Smoking		
No	Reference	
Yes	0.85 (0.64–1.14)	.233
Drinking	1.07 (0.52–2.20)	.86
No	Reference	
Yes	0.91 (0.66–1.24)	.537
SBP	1.01 (1.00–1.01)	.285
DBP	1.01 (1.00–1.01)	.164
BMI	1.01 (0.96–1.04)	.912

Variables with normal distribution were presented as mean ± standard deviation, other variables with classification were described as counts (percentages).

Discrepant *P*-values are represented in bold.

AF = atrial fibrillation, ALT = Alaninetransaminase, Apo A1 = Apolipoprotein A1, Apo B = Apolipoprotein B, AST = aspartate amino transferase, BMI = body mass index, DBP = diastolic blood pressure, FBG = fasting blood glucose, HBA1c = hemoglobin A1c, HDL-C = high-density lipoprotein cholesterol, hsCRP = highly sensitive C-reactive protein, LDH = lactate dehydrogenase, LDL-C = low-density lipoprotein cholesterol, LVEF = left ventricular ejection fraction, LVFS = left ventricular fractional shortening, RBC = red blood cell, SBP = systolic blood pressure, sCD40L = soluble CD40 ligand, TG = triglyceride, WBC = white blood cell.

**Figure 3.**
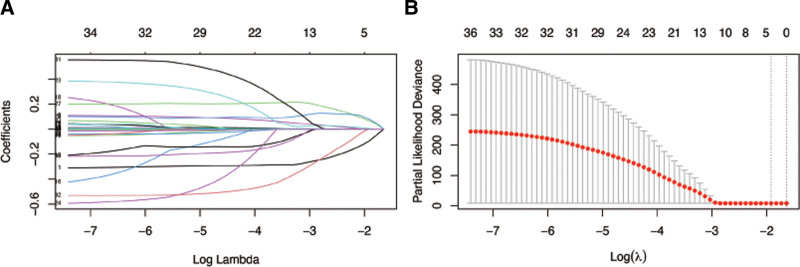
(A) LASSO coefficient profiles of the 34 risk factors. (B) Risk factors selected using LASSO regression analysis. LASSO = The Least Absolute Shrinkage and Selection Operator.

**Figure 4.**
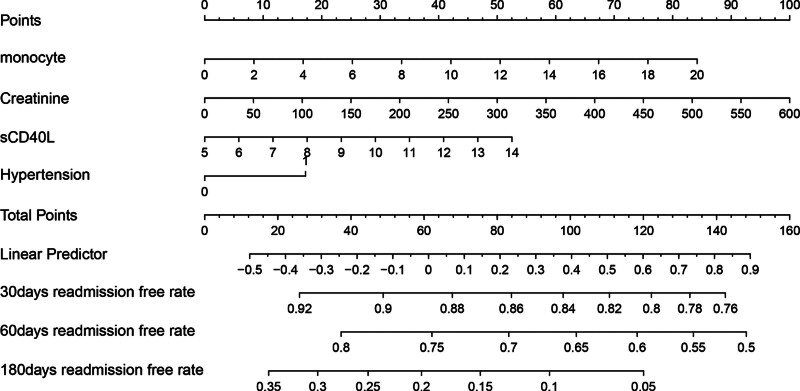
Developed a new nomogram for readmission within 180 days for patients with chronic heart failure.

**Figure 5.**
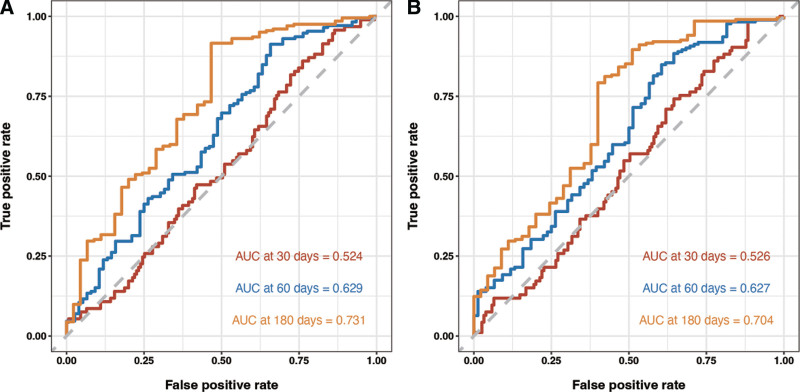
ROC curves from training dataset (A) and validation dataset (B) using nomogram. ROC = receiver operator characteristic.

**Figure 6.**
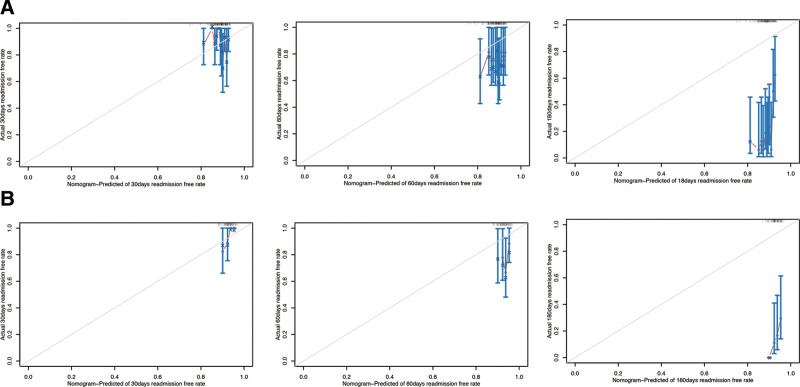
The calibration curve represents the difference between the actual prediction (blue line) and the ideal perfect prediction (45° line). (A) 30 days, 60 days, and 180 days readmission-free rate in the training dataset of nomogram. (B) 30 days, 60 days, and 180 days readmission-free rate in the validation dataset of nomogram.

**Figure 7.**
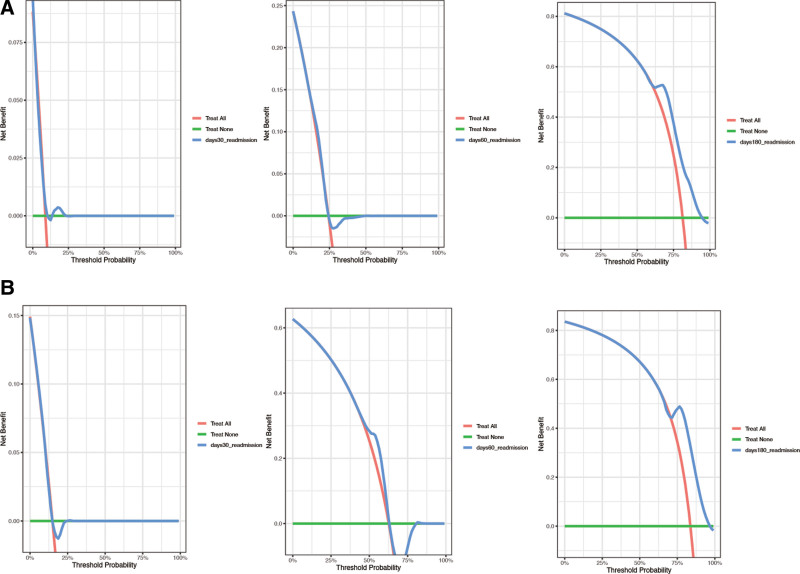
Decision curves for the nomogram in training dataset (A) and validation dataset (B)

Subsequently, we divided the dataset into training dataset and validation dataset. As shown in Table S1, Supplemental Digital Content, http://links.lww.com/MD/O665, most features had no statistical differences. We then used LASSO COX regression to identify the best feature variables, selecting 4 out of 34 variables (Fig. 3) in training dataset. We considered monocytes (OR: 1.04; 95% CI: 1.00–1.09; *P* = .051), serum creatinine (OR: 1.02; 95% CI: 1.00–1.02; *P* = .042), sCD40L (OR: 1.07; 95% CI: 1.00–1.14; *P* = .033), and a history of hypertension (OR: 1.30; 95% CI: 1.01–1.58; *P* = .048) as independent risk factors for readmission in patients with chronic HF (Table 4). We then constructed a nomogram based on these features to predict the probability of readmission in patients with chronic HF within 30 days, 60 days, and 180 days (Fig. 4). The nomogram showed the best predictive ability of the model for readmission at 6 months, with AUC of 0.524, 0.629, and 0.731 in the training dataset respectively and the area in the validation dataset was 0.526, 0.627, and 0.704 at 30, 60, and 180 days, respectively (Fig. 5). To evaluate the consistency between actual readmission and predicted probability, we plotted a calibration curve, as shown in Figure 6. In both the training and validation datasets, the predicted and actual readmission of the model remained relatively consistent. To evaluate the clinical effectiveness of the model, we conducted a decision curve analysis, which showed good net benefit under the probability of column line events in both the training and validation dataset, with higher net benefit in the validation dataset (Fig. 7).

**Table 4:** LASSO COX regression analysis.

Variable	Hazard radio (95% CI)	*P*-value
Monocyte	1.04 (1.00–1.09)	.051
Creatinine	1.02 (1.00–1.02)	**.042**
sCD40L	1.07 (1.00–1.14)	**.023**
Hypertension		
No	Reference	
Yes	1.30 (1.01–1.59)	**.048**

Discrepant *P*-values are represented in bold.

LASSO = The Least Absolute Shrinkage and Selection Operator, sCD40L = soluble CD40 ligand.

### 3.4. Model performance

**Figure 8.**
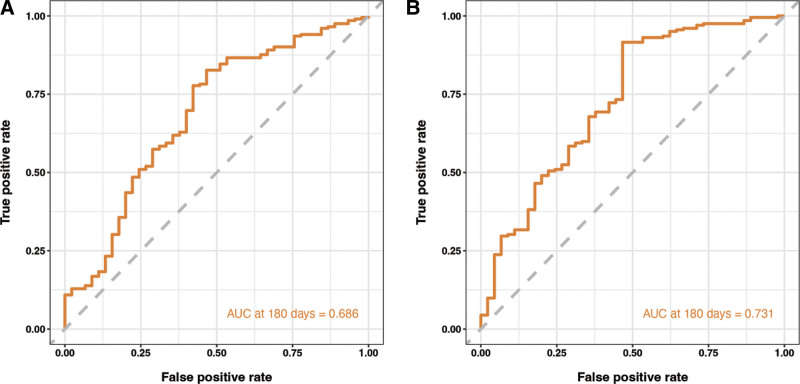
(A) ROC curves of model with monocytes, creatinine, and hypertension history. (B) ROC curves of model with monocytes, creatinine, and hypertension history and sCD40L. ROC = receiver operator characteristic, sCD40L = soluble CD40 ligand.

In Figure 8, we present the AUC for different models or parameters. Our results show that when monocytes, creatinine, and a history of hypertension were used to model readmission in patients with chronic HF, the AUC was 0.686. However, when sCD40L was added to this model, the AUC significantly improved to 0.731, indicating a higher accuracy in predicting readmission.

## 4. Discussion

In this study, we examined the relationship between sCD40L and readmission in patients with chronic HF, as well as its association with biochemical, demographic, and imaging data. Notably, our study is the first to establish a link between sCD40L levels and readmission in patients with chronic HF. Using LASSO analysis, we identified monocytes, creatinine, sCD40L, and hypertension history as independent risk factors for readmission in these patients. Additionally, we developed a nomogram based on sCD40L to predict the probability of readmission for chronic HF.

The clinical features of HF patients requiring readmission have been explored, and studies have shown that hemoglobin, blood sodium, SBP, heart rate, blood urea nitrogen, age, troponin, and brain natriuretic peptide are risk factors for readmission within 30 days.^[11]^ Gao study included 5 variables, acute HF, emergency department visit, age, blood urea nitrogen level, and beta-blocker usage, resulting in AUC of 0.75 for their final model.^[12]^ Our results are slightly lower than Gao’s, which could be attributed to the fact that our study focused on chronic HF patients, whereas Gao study included both chronic and acute HF cases. Similarly, Zheng conducted a related study using a public database to establish a prediction model for readmission in HF patients.^[13]^ However, Zheng model only included variables related to patient comorbidities and complications, excluding laboratory markers, which resulted in AUC values of 0.58 for the training set and 0.59 for the validation set. Earlier studies have constructed predictive models for the readmission of HF patients, with age, New York Heart Association, body mass index, blood creatinine, and inflammatory markers as key factors, and the AUC of the model reached 0.739 (95% CI: 0.673–0.800).^[14]^ Studies have also used LASSO regression to screen for risk factors of readmission within 180 days in HF patients. Age, acute HF, emergency, beta-blocker usage, and blood urea nitrogen were ultimately included in the criteria, and the model’s AUC reached 0.75 (95% CI: 0.69–0.81).^[12]^ In our model, the AUC was 0.686 without the use of sCD40L. However, when sCD40L was included as a predictive factor, the AUC reached 0.731, indicating a significant improvement in the prediction.

sCD40L, an inflammatory mediator, is mainly released by activated platelets^[6]^ and is a strong predictor of cardiovascular events. In animal models, previous studies have shown that CD40L promotes platelet aggregation, monocyte recruitment, and exosmosis by promoting the formation of ultra-large von Willebrand factor multipliers, leading to atherosclerosis.^[15]^ In a myocardial infarction model, recombinant sCD40L can specifically bind alphaIIbbeta3 and activated platelets, inducing platelet diffusion.^[16]^ Some studies have explored the relationship between cardiovascular disease and sCD40L levels. In a study by Christopher, an increase in sCD40L levels in acute coronary syndrome leads to an increased risk of recurrent myocardial infarction and death.^[17]^ Hamdi also obtained similar results, indicating that higher levels of sCD40L were associated with the 1-year all-cause mortality rate of ST-segment elevation myocardial infection in patients receiving primary percutaneous coronary intervention.^[18]^ Recent studies have shown that changes in the c allele of CD40’s single nucleotide polymorphism can cause a pro-inflammatory phenotype in endothelial cells.^[19]^ In summary, several studies have confirmed that sCD40L is closely associated with the risk of cardiovascular diseases. Therefore, further research is required to confirm the relationship between sCD40L levels and cardiovascular disease prognosis.

We also conducted a correlation analysis between sCD40L levels and various parameters. Our findings revealed a positive correlation between sCD40L and males, which is consistent with existing literature. This may be due to the higher smoking rate among men, as smoking is a key factor leading to inflammatory reactions.^[20,21]^ Additionally, we observed a positive correlation between sCD40L and some inflammatory markers and harmful metabolites such as high-sensitivity c-reactive protein, uric acid, and low-density lipoprotein cholesterol, which is in line with previous reports. However, we found a negative correlation between sCD40L levels and fasting blood glucose, HBA1C, diabetes history. This could be because we did not collect information on the medication of patients for controlling blood sugar levels. Moreover, we noted a positive correlation between sCD40L levels and LVEF/LVFS. These are indicators used to diagnose HF and reflect its severity. Taken together, our results suggest that high sCD40L levels are associated with readmission risk in patients with chronic HF.

Monocytes are important immune cells, and existing studies have shown that the levels of inflammatory cytokines are significantly elevated in patients with HF. Monocytes play a crucial role in the inflammatory cascade and are a major source of both pro-inflammatory and anti-inflammatory factors.^[22]^ Additionally, monocytes can differentiate into macrophages, further exacerbating inflammation and promoting the progression of HF.^[23]^ Furthermore, monocytes are associated with a range of chronic diseases, such as chronic obstructive pulmonary disease^[24,25]^ and cardiovascular diseases.^[26]^ Studies have found that elevated monocyte levels are correlated with higher rates of readmission and mortality in cardiovascular patients.^[26,27]^ We believe that an increase in monocytes typically reflects an escalation in both cardiac and systemic inflammatory responses, which may lead to further cardiovascular damage and remodeling, exacerbating the symptoms of HF. High monocyte levels could signal an acute worsening of HF, thereby increasing the risk of readmission.

Creatinine is an important marker of kidney function, and renal impairment is closely related to the pathology of HF. Chronic kidney disease and HF often coexist, with kidney dysfunction worsening the symptoms of HF, while HF also exacerbates kidney dysfunction. Creatinine is routinely used to assess the severity and prognosis of the disease.^[28,29]^ We hypothesize that elevated creatinine levels usually indicate renal insufficiency, which can lead to fluid retention and electrolyte imbalances, thereby worsening the course of HF. Therefore, an increase in creatinine levels is an important indicator of readmission risk for HF patients.

Hypertension is one of the most common triggers for HF. Long-term hypertension increases the burden on the heart, particularly leading to left ventricular hypertrophy, ventricular remodeling, and eventually HF. A history of hypertension suggests that the patient may have experienced prolonged cardiac stress and vascular damage, which increases the risk of worsening HF. For patients with a history of hypertension, the progression of HF is often more complex and difficult to treat, thus raising the likelihood of readmission.

Finally, we constructed a column chart based on sCD40L to predict the risk of readmission within 180 days for patients with chronic HF. Our model has demonstrated good accuracy and can provide clinicians with valuable insights to develop a more comprehensive plan for managing HF patients.

Our study had some limitations. First, sCD40L levels were measured from the blood samples collected from patients 2 to 3 days after admission. During sample processing, there may be degradation of sCD40L, which may cause errors in the experimental results. Second, although this was a prospective cohort study, there was no causal relationship between sCD40L levels in chronic HF patients and the time of readmission. Finally, due to the limitations of this study, our sample size is relatively small, and we were unable to conduct multi-center validation, which may affect the generalizability and applicability of the model’s results. Although we made efforts to ensure the quality and representativeness of the data, a single-center study still has certain limitations and may not fully reflect the clinical characteristics of different regions or populations. Therefore, future studies need to expand the sample size and consider conducting multi-center validation to improve the reliability and accuracy of the model.

## 5. Conclusion

The level of sCD40L was related to readmission results in HF patients. Patients with high sCD40L levels had an increased risk of readmission within 180 days and exhibited significant associations with monocytes, creatinine, and a history of hypertension. Consequently, the model has good predictive performance in stratifying chronic patients HF and in timely clinical decision making.

## Author contributions

**Conceptualization:** Peng Zhang.

**Data curation:** Peng Zhang, Xiaoya Zhai, Yefei Gao.

**Formal analysis:** Peng Zhang, Xiaoya Zhai.

**Funding acquisition:** Liping Meng, Hui Lin, Ping Wang, Chengjian Jiang.

**Investigation:** Peng Zhang, Yefei Gao, Chengjian Jiang.

**Methodology:** Peng Zhang, Ping Wang, Chengjian Jiang.

**Project administration:** Peng Zhang, Yefei Gao, Liping Meng, Hui Lin.

**Resources:** Yefei Gao, Liping Meng.

**Software:** Xiaoya Zhai, Yefei Gao, Liping Meng, Hui Lin, Ping Wang.

**Supervision:** Xiaoya Zhai, Yefei Gao, Liping Meng, Chengjian Jiang.

**Validation:** Xiaoya Zhai, Yefei Gao, Ping Wang.

**Visualization:** Peng Zhang, Xiaoya Zhai, Hui Lin, Ping Wang.

**Writing – original draft:** Peng Zhang, Xiaoya Zhai.

**Writing – review & editing:** Liping Meng, Hui Lin.

## Supplementary Material


