# Supplementary material for: Construction and evaluation of a 180-day readmission prediction model for chronic heart failure patients based on sCD40L
Source: Medicine (Baltimore). 2025 Apr 11;104(15):e42134. doi: 10.1097/MD.0000000000042134 (PMC11999405; doi:10.1097/MD.0000000000042134)
Supplement: Supplementary file 1 [file medi-104-e42134-s001.docx]

TableS1 Baseline characteristics of patients

| sex: | Training dataset (N=166) | Validation dataset (N=82) | p value |
| --- | --- | --- | --- |
| Female | 53 (31.9%) | 31 (37.8%) | 0.437 |
| Male | 113 (68.1%) | 51 (62.2%) |  |
| age, years | 57.1 (12.2) | 59.2 (13.1) | 0.238 |
| WBC, *1012/L | 5.44 (1.86) | 5.52 (2.28) | 0.769 |
| Neutrophils,*1012/L | 6.06 (6.53) | 5.70 (7.22) | 0.708 |
| lymphocyte,*1012/L | 4.95 (11.6) | 3.95 (8.17) | 0.436 |
| monocyte,*1012/L | 2.70 (3.26) | 1.93 (2.33) | 0.065 |
| RBC, *1012/L | 3.21 (2.17) | 3.66 (2.44) | 0.164 |
| hsCRP, mg/L | 3.86 (8.40) | 3.41 (6.85) | 0.655 |
| AST, U/L | 143 (1464) | 28.5 (14.7) | 0.315 |
| LDH, U/L | 329 (1230) | 230 (77.9) | 0.304 |
| Albumin, g/L | 37.1 (5.16) | 37.9 (4.25) | 0.181 |
| Globulin, g/L | 29.7 (4.55) | 28.3 (4.48) | 0.026 |
| Creatinine, umol/L | 99.0 (84.8) | 84.9 (24.9) | 0.049 |
| Urea, umol/L | 412 (137) | 418 (122) | 0.729 |
| TG, mmol/L | 1.30 (0.72) | 1.25 (0.65) | 0.585 |
| HDL-C, mmol/L | 1.06 (0.30) | 1.08 (0.29) | 0.556 |
| LDL-C, mmol/L | 2.52 (0.81) | 2.50 (0.72) | 0.802 |
| Apo A1, g/L | 1.09 (0.27) | 1.12 (0.27) | 0.536 |
| Apo B, g/L | 0.87 (0.27) | 0.83 (0.24) | 0.256 |
| FBG, mmol/L | 6.94 (3.68) | 6.29 (2.28) | 0.091 |
| ALT, U/L | 61.3 (432) | 32.5 (30.6) | 0.394 |
| HBA1c, (%) | 6.87 (1.81) | 6.69 (1.53) | 0.394 |
| sCD40L, ng/ml | 9.55 (2.08) | 9.51 (2.15) | 0.878 |
| LVEF, % | 49.9 (13.9) | 49.9 (13.5) | 0.997 |
| LVFS, % | 26.5 (8.84) | 26.1 (8.24) | 0.693 |
| AF |  |  | 0.395 |
| No | 94 (56.6%) | 41 (50.0%) |  |
| Yes | 72 (43.4%) | 41 (50.0%) |  |
| Hypertension: |  |  | 0.686 |
| No | 85 (51.2%) | 39 (47.6%) |  |
| Yes | 81 (48.8%) | 43 (52.4%) |  |
| hyperlipidemia: |  |  | 0.504 |
| No | 158 (95.2%) | 80 (97.6%) |  |
| Yes | 8 (4.82%) | 2 (2.44%) |  |
| diabetes: |  |  | 0.561 |
| No | 107 (64.5%) | 49 (59.8%) |  |
| Yes | 59 (35.5%) | 33 (40.2%) |  |
| smoking: |  |  | 0.484 |
| No | 120 (72.3%) | 55 (67.1%) |  |
| Yes | 46 (27.7%) | 27 (32.9%) |  |
| drinking: |  |  | 0.494 |
| No | 130 (78.3%) | 68 (82.9%) |  |
| Yes | 36 (21.7%) | 14 (17.1%) |  |
| SBP,mmhg | 131 (21.6) | 133 (20.9) | 0.481 |
| DBP,mmhg | 80.8 (13.8) | 80.7 (14.6) | 0.956 |
| BMI (kg/m2) | 23.7 (3.48) | 24.3 (3.71) | 0.29 |
| day | 136 (116) | 123 (103) | 0.361 |

WBC, white blood cell; RBC, red blood cell; hsCRP, highly sensitive C-Reactive Protein; AST, aspartate amino transferase; LDH, lactate dehydrogenase; TG, triglyceride; HDL-C, high-density lipoprotein cholesterol; LDL-C, low-density lipoprotein cholesterol; Apo A1; Apolipoprotein A1; Apo B, Apolipoprotein B; FBG, fasting blood glucose; ALT, Alaninetransaminase; HBA1c, hemoglobin A1c; LVEF, left ventricular ejection fraction; LVFS, left ventricular fractional shortening; AF, atrial fibrillation; SBP, systolic blood pressure; DBP, diastolic blood pressure; BMI, Body mass index. Discrepant p-values are represented in bold.
